# Dietary inflammatory index and non-alcoholic fatty liver disease risk: a systematic review and meta-analysis of observational studies

**DOI:** 10.3389/fnut.2025.1596300

**Published:** 2025-06-20

**Authors:** Bahareh Amirkalali, Mohammad Farahmand, Minoo Hasan Rashedi, Ali Gholami, Fateme Sheikholmolooki, Mohadeseh Sedighi, Azam Doustmohammadian

**Affiliations:** ^1^Gastrointestinal and Liver Diseases Research Center, Iran University of Medical Sciences, Tehran, Iran; ^2^Pediatric Infectious Disease Research Center, Tehran University of Medical Sciences, Tehran, Iran; ^3^Department of Nutrition, School of Public Health, Iran University of Medical Sciences, Tehran, Iran; ^4^Noncommunicable Diseases Research Center, Neyshabur University of Medical Sciences, Neyshabur, Iran; ^5^Workplace Health Research Center, Neyshabur University of Medical Sciences, Neyshabur, Iran; ^6^Department of Nutrition, Health and Statistics Surveillance Research Center, Science and Research Branch, Islamic Azad University, Tehran, Iran; ^7^Department of Internal Medicine, School of Medicine, Firoozgar General Hospital, Iran University of Medical Sciences, Tehran, Iran

**Keywords:** non-alcoholic fatty liver disease, inflammation, diet, observational studies as topic, meta-analysis

## Abstract

**Aim:**

Chronic inflammation plays a significant role in the progression of non-alcoholic fatty liver disease (NAFLD). Adopting an anti-inflammatory diet can help prevent or mitigate NAFLD and its associated complications. This meta-analysis builds on previous research by examining the association between the Dietary Inflammatory Index (DII) and NAFLD risk, incorporating additional studies and employing rigorous evidence assessment.

**Methods:**

We systematically searched major databases (Cochrane Library, PubMed, Web of Science, and Scopus) from inception to June 2024 for English-language observational studies examining the association between DII and NAFLD prevalence. Pooled odds ratios (ORs) or hazard ratios (HRs) with 95% confidence intervals (CIs) were calculated using random-effects models for studies with significant heterogeneity; otherwise, fixed-effects models were applied. Subgroup analyses were conducted to explore heterogeneity based on body mass index (BMI), DII definition, sample size, geographical region, age, and NAFLD diagnostic criteria. Evidence certainty was assessed using the Grading of Recommendations, Assessment, Development, and Evaluation (GRADE) framework. The study was registered in PROSPERO (CRD42023430798).

**Results:**

Eleven studies (9 cross-sectional with 14 effect sizes and 2 cohort with 2 effect sizes) were analyzed. Higher DII scores were significantly associated with increased NAFLD risk, with a pooled OR of 1.56 (95% CI: 1.24–1.95; *p* < 0.001) in cross-sectional studies and an HR of 0.21 (95% CI: 0.12–0.30; *p* < 0.0001) in cohort studies. Subgroup analyses confirmed consistency across BMI ≥ 25, energy-adjusted DII or DII, studies in Asia and Europe, and participants <46 years, with reduced heterogeneity (I^2^ < 50%) in these categories. GRADE rated the certainty of evidence as “very low.”

**Conclusion:**

Anti-inflammatory diets can reduce NAFLD risk. However, high-quality studies are needed to confirm this association.

## 1 Introduction

Non-alcoholic fatty liver disease (NAFLD) is a major global health concern, affecting approximately 25% of adults worldwide and imposing a significant economic burden (1, 2). NAFLD is defined as excessive hepatic fat accumulation detected via imaging or biopsy after excluding causes such as excessive alcohol consumption, hepatotoxic medications, toxins, viral infections, or genetic liver disorders (3). The condition spans a spectrum from simple steatosis to non-alcoholic steatohepatitis (NASH), which can lead to fibrosis, cirrhosis, and hepatocellular carcinoma (4).

Chronic inflammation is a key driver of NAFLD pathogenesis and progression (5, 6). In NAFLD, hepatocytes, stressed by lipotoxicity, and immune cells, such as Kupffer cells, release pro-inflammatory cytokines, including tumor necrosis factor-alpha (TNF-*α*), interleukin-6 (IL-6), C-reactive protein (CRP), and interleukin-1β (IL-1β), which amplify hepatic inflammation (7, 8). These cytokines promote fibrogenesis, causing progression of NAFLD from steatosis to severe liver disease (7). Lifestyle modifications and targeted therapies that address inflammation offer promising strategies to prevent NAFLD progression (9, 10).

Diet plays a critical role in modulating inflammation and reducing NAFLD risk (11, 12). Diets rich in fruits, vegetables, whole grains, and fiber are inversely associated with inflammatory biomarkers, while diets high in refined grains, sweetened beverages, processed meats, and added fats are positively linked to inflammation (13–15). The Dietary Inflammatory Index (DII) was developed based on research linking dietary patterns to inflammatory biomarkers, including pro-inflammatory factors such as CRP, IL-1β, IL-6, and TNF-*α*, which exacerbate hepatic inflammation and promote NAFLD progression, as well as anti-inflammatory markers such as interleukin-4 (IL-4) and interleukin-10 (IL-10) (16, 17). This index quantifies the inflammatory potential of diets by assigning scores from −8.87 (highly anti-inflammatory) to +7.98 (highly pro-inflammatory) based on 45 food components (17). Anti-inflammatory foods (e.g., fatty fish, nuts, and fruits) receive lower scores, while pro-inflammatory foods (e.g., processed meats and sugary drinks) receive higher scores (17). Higher DII scores are associated with an increased risk of chronic diseases, including cardiovascular disease, diabetes, and NAFLD, underscoring the importance of dietary choices (16, 17).

Despite evidence linking DII to NAFLD (18–21), prior studies are limited by inconsistent designs, small sample sizes, and inadequate confounder adjustment, resulting in uncertain evidence. Qianwen Zhao’s meta-analysis (22) reported a positive association between higher DII scores and increased NAFLD risk but was limited by the exclusion of four high-quality observational studies (18, 20, 23, 24) and the lack of evidence certainty assessments. Our meta-analysis overcomes these limitations by including the four studies (18, 20, 23, 24) and applying the Grading of Recommendations, Assessment, Development, and Evaluation (GRADE) framework to evaluate evidence certainty, resulting in a more robust and precise understanding of the relationship between dietary inflammation and NAFLD risk in adults.

## 2 Methods

### 2.1 Search strategy

The meta-analysis was carried out in accordance with the Preferred Reporting Items for Systematic Reviews and Meta-Analyses (PRISMA) guidelines (Supplementary Table S1) (25), and the protocol was registered with PROSPERO (CRD42023430798, Available at: https://www.crd.york.ac.uk/PROSPERO/display_record.php?ID=CRD42023430798). We searched electronic databases, including the Cochrane Library, PubMed, Web of Science, and Scopus, for publications regarding DII and NAFLD, up to December 2024, with no time limit. The literature on DII and NAFLD was searched using the following keywords: dietary inflammatory index (DII), NAFLD, fatty liver, hepatic steatosis, liver steatosis, and steatohepatitis. The search strategy was created using Boolean operators, asterisks, quotation marks, and parentheses. The database-specific search strategies are detailed in Supplementary Table S3. Subsequently, all identified papers were uploaded to the reference management software (EndNote X20, Clarivate Analytics, United States) to coordinate the review process, remove duplicates, and manage citations. To identify relevant articles, three reviewers (AD, FS, and MS) independently evaluated the titles and abstracts of all papers, and further the full text of the eligible studies was appraised. The articles that met the inclusion criteria were kept for data extraction. Additionally, the reference lists of the included articles were reviewed to ensure the thoroughness of searches.

### 2.2 Study eligibility criteria

The inclusion of a study was guided by the Population, Intervention /Exposure, Comparator, Outcome, and Study (PICOS) framework. Eligible studies were required to meet all the following criteria:

a Study design: Observational studies (cross-sectional, case–control, or cohort) conducted in adults aged ≥18 years.
b Intervention/Exposure: Dietary inflammatory potential assessed using the DII score.
c Outcome: NAFLD, diagnosed via liver ultrasound, fatty liver index (FLI), controlled attenuation parameter (CAP), hepatic steatosis index (HSI), or magnetic resonance imaging (MRI).
d Effect estimates: Reported multivariable-adjusted odds ratios (ORs) or hazard ratios (HRs), with corresponding 95% confidence intervals (CIs), comparing the highest (pro-inflammatory) to the lowest (anti-inflammatory) DII categories.
e Language: Full-text articles published in English.

Studies were excluded if they:

a included participants with secondary hepatic steatosis (e.g., drug-induced), viral hepatitis, cirrhosis, or those receiving enteral or parenteral nutrition;
b were animal studies;
c were non-original research (e.g., reviews, editorials, and conference abstracts) or duplicate publications.

### 2.3 Data extraction

Three researchers (AD, FS, and BA) independently extracted the following information from the list of included eligible studies: first author’s last name, year of publication, geographic region, study design, sample size, gender distribution, mean age, the method used for NAFLD diagnosis, number of food parameters for DII calculation, DII score range, type of DII data and comparison level, adjusted total effect estimates and their corresponding 95% confidence intervals (CIs), and adjusted confounding factors.

Discrepancies, if any, were discussed and resolved by consensus and arbitration with the research team or experts in the field. If there was any confusion regarding the information, an email was sent to the corresponding author.

### 2.4 Quality and risk of bias assessment

The quality of eligible studies was evaluated using the Newcastle–Ottawa scale (NOS) (26). This scale consists of three categories: selection, comparability, and exposure. Each component in the selection and exposure categories can only receive one point, but the comparison can receive up to two points. A score of 7 or higher indicated high quality (27). The risk of bias assessment was conducted by using the Risk Of Bias In Non-randomized Studies of Interventions (ROBINS-I) tool in the observational literature (28). The ROBINS-I tool assesses bias based on seven domains, including:

a confounding
b selection of study participants
c classification of exposures
d departure from intended exposure
e missing data
f outcome measurement, and
g selection of reported results.

Studies were categorized as having a low, moderate, serious, or critical risk of bias under each domain. The quality and risk of bias of eligible studies were evaluated separately by two researchers (BA and FS), and the discrepancy, if any, was resolved after a discussion with the principal investigator.

### 2.5 Evaluating the certainty of the evidence

The level of certainty in the evidence for the main outcomes was evaluated by utilizing the Grading of Recommendations, Assessment, Development, and Evaluation (GRADE) tool (29). The assessment considered factors including risk of bias, inconsistency, indirectness, and imprecision (Table 1).

**Table 1:** Certainty of evidence of primary outcomes using the GRADE tool.

Certainty assessment							№ of patients		Effect		Certainty	Importance
№ of studies	Study design	Risk of bias	Inconsistency	Indirectness	Imprecision	Other considerations	Cases	Non-cases	Relative (95% CI)	Absolute (95% CI)		
Cohort												
Ultrasound												
1	NRS	Not serious	Not serious	Serious^a^	Not serious	None	2,744	12,877	**HR 1.26** (1.13 to 1.41)	**1 fewer per 1,000** (from 1 fewer to 1 fewer)	⨁◯◯◯ Very low^a^	IMPORTANT
MRI												
1	NRS	Not serious	Not serious	Serious^a^	Not serious	None	1,489	171,544	**HR 1.19** (1.03 to 1.38)	**1 fewer per 1,000** (from 1 fewer to 1 fewer)	⨁◯◯◯ Very low^a^	IMPORTANT
Cross-sectional												
FLI												
8	NRS	Not serious	Very serious^b^	not serious	Not serious	Publication bias strongly suspected^c^	32,541	31,956	**OR 1.21** (1.17 to 1.24)	**0 fewer per 1,000** (from 0 fewer to 0 fewer)	⨁◯◯◯ Very low^b,c^	IMPORTANT
HS												
3	NRS	Not serious	Serious^d^	Not serious	Not serious	None	8,810	6,710	**OR 1.13** (1.09 to 1.17)	**1 fewer per 1,000** (from 1 fewer to 1 fewer)	⨁◯◯◯ Very low^d^	IMPORTANT
CAP												
2	NRS	Not serious	Serious^d^	Not serious	Serious^e^	None	133	67	**OR 1.12** (0.88 to 1.42)	**1 fewer per 1,000** (from 1 fewer to 1 fewer)	⨁◯◯◯ Very low^d,e^	IMPORTANT
Ultrasound												
1	NRS	Not serious	Not serious	Serious^a^	Not serious	None	3,110	1,452	**OR 1.54** (1.23 to 1.93)	**2 fewer per 1,000** (from 2 fewer to 1 fewer)	⨁◯◯◯ Very low^a^	IMPORTANT

CI, confidence interval; HR, hazard ratio; OR, odds ratio; NRS, non-randomized study. Explanation. ^a^Because of the singular study. ^b^Because of the severe heterogeneity. ^c^Because of the Egger’s test. ^d^Because of the moderate heterogeneity. ^e^Because of the non-significant result.

### 2.6 Statistical analysis

A meta-analysis was conducted utilizing statistical software, Stata version 12.0. Risk estimates from various studies were combined using either a random-effects model or a fixed-effects model to calculate the pooled ORs with 95% CIs. Heterogeneity across studies was assessed based on the Cochrane Q test and I^2^ statistic. The level of heterogeneity was assessed based on the I^2^ statistic, with values of 25, 50, and 75% indicating low, moderate, and high heterogeneity, respectively. Significant heterogeneity was set at a *p*-value of <0.1 for the Cochrane Q test or I^2^ statistic >50%. A random-effects model was applied when pooled analysis resulted in statistical heterogeneity, and a fixed-effects model was used otherwise. Subgroup analyses were carried out based on the sample size (≤3,042, >3,042), age (<46 years, ≥46 years), BMI (<25, ≥25), study design (cross-sectional, cohort), study regions (Asia, America, Europe), type of DII (energy-adjusted DII (E-DII), DII), and NAFLD diagnosis method (HSI, FLI, CAP, ultrasonography) to investigate the sources of heterogeneity (30, 31).

## 3 Results

### 3.1 Literature search and included studies

**Figure 1:**
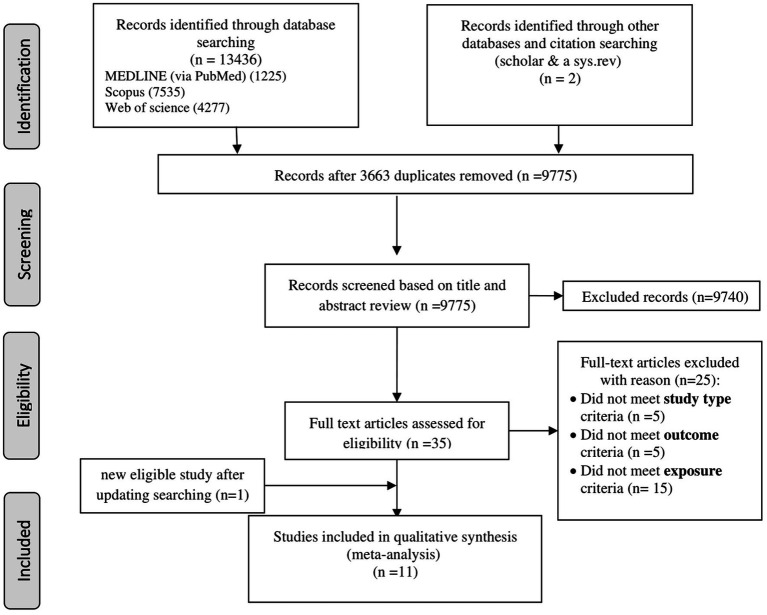
Flowchart of the literature search and selection.

A systematic search of six databases and citation searches yielded 13,438 articles, with 3,663 duplicates excluded (Figure 1). After screening 9,775 titles and abstracts, 9,740 irrelevant articles (e.g., reviews, non-human studies, congress abstracts, and study protocols) were excluded. Of the 35 full-text articles assessed, 24 were excluded for not meeting the inclusion criteria (e.g., study type, outcome, and exposure; Supplementary Table S2). An updated search added one eligible study (19), resulting in 11 included articles: 9 cross-sectional (18–21, 23, 32–35) and 2 cohort studies (36, 37). Three studies (18, 20, 23) reported 2 NAFLD diagnostic methods, while 1 study (24) reported 3, resulting in 14 effect sizes for cross-sectional studies and 2 for cohort studies, which were analyzed separately.

### 3.2 Study characteristics

The 9 cross-sectional studies included 82,974 participants from the USA [5 studies; (18–20, 32, 35)], Iran [3 studies; (21, 33, 34)], and Greece [1 study; (23)], using various NAFLD diagnostic tools including FLI (8 studies), HSI (3 studies), liver ultrasonography (1 study), and CAP (1 study). Dietary inflammation was assessed via E-DII (4 studies) or DII (5 studies), with ORs comparing the highest and lowest DII quartiles (4 studies) or tertiles (5 studies). The two cohort studies, involving 184,421 participants, assessed dietary inflammation and NAFLD using E-DII with MRI in the United Kingdom (37) and DII with liver ultrasonography in China (36) (Table 2).

**Table 2:** Data extracted from the studies included for meta-analysis.

	Author, year	Study location	Study design	Study period	Instrument for NAFLD detection	Number of food parameters	Sample size	Range DII score	Type of data and comparison/level of comparison	Measures of association	Adjustment factors
1	Rui and Lin (18)	USA	Cross-sectional	2011 to 2018	1) HSI 2) US. FLI > 30	E-DII:33	12,410	−7.60 to 6.98	Categorical (quartile 1 vs. quartile 4)	HSI: OR = 0.77 (95% CI: 0.62–0.96) USFLI: OR = 0.48 (95% CI: 0.35–0.68)	Age, sex, BMI, education level, physical activity, smoking, SES (household income), E-DII, ethnicity, marital status, household income, and total cholesterol concentration
2	Mazidi et al. (19)	USA	Cross-sectional	2006–2012	_US_. FLI ≥ 30	E-DII:45	20,643	−5.66 to 4.24	Categorical (quartile 4 vs. quartile 1)	OR = 5.97 (95% CI: 4.44–8.02)	Age, sex, physical activity, smoking, alcohol consumption, SES, energy intake, and HDL levels.
3	Ramírez-Vélez et al. (20)	USA	Cross-sectional	2017–2018	1) FLI 2) CAP≥ 233 dB m–1	DII: 26	4,189	−1.531 ± 0.795 to 1.593 ± 0.488	Categorical (tertile 1 vs. tertile 3)	FLI: OR = 0.722 (95% CI: 0.537–0.972) CAP: OR = 0.952 (95% CI: 0.749–1.211)	Age, sex, BMI, physical activity, smoking, alcohol consumption, energy intake, HDL levels, race, and citizenship status
4	Soltanieh et al. (21)	Iran	Cross-sectional		CAP≥270 db/m	E-DII:31	200	< − 3.11 to > − 2.49	Categorical (tertile 3 vs. tertile 1)	OR = 2.78 (95% CI: 1.09–7.13)	Age, sex, BMI, physical activity, smoking, SES, energy intake, serum fasting blood sugar (continuous), serum triglyceride (continuous), serum cholesterol (continuous), and HOMA (continuous).
5	Tyrovolas et al. (23)	Greece	Cross-sectional	2001–2002	1) FLI 2) HSI > 36	D-AII: 45 food items (according to the methodology by Shivappa et al.)	3,042	NS	Categorical (tertile 3 vs. tertile 1)	FLI: OR = 0.88 (95% CI: 0.85–0.91) HSI: OR = 0.89 (95% CI: 0.86–0.92)	Age, sex, BMI (% obesity), education level, physical activity, smoking, alcohol consumption, SES, and WHR.
6	Zhang et al. (32)	USA	Cross-sectional	2005–2016	FLI score ≥60	E-DII: NS/24 h food recall	10,052	NS	Categorical (quartile 4 vs. quartile 1)	OR = 1.52 (95% CI: 1.27–1.83)	Sex, BMI, education level, smoking, SES (poverty income ratio), race, marital status, waist circumference, AST, ALT, and GGT.
7	Valibeygi et al. (33)	Iran	Cross-sectional	2013–2019	FLI cutoff ≥ 60	EDII: 32/ 168-item FFQ	9,792	NS	Categorical (tertile 3 vs. tertile 1)	OR = 1.254 NS	Age, sex, BMI, physical activity, energy intake, hypercholesterolemia, hypertriglyceridemia, white blood cell, ALP, and GGT.
8	Doustmohammadian et al. (24)	Iran	Cross-sectional	2017	1) Ultrasound 2) FLI 3) HSI	EDII: 32/168-item FFQ	3,110	NS	Categorical (tertile 3 vs. tertile 1)	Ultrasound OR = 1.54 (95%CI: 1.23–1.93) FLI: OR = 1.78 (95%CI: 1.28–2.47) HSI: OR = 1.43 (95%CI: 1.11–1.85)	Age, physical activity, smoking, alcohol consumption, WHR, energy intake, serum lipid-lowering drugs, HPTN-lowering drugs, lowering serum glucose drugs, residual areas, family history of diabetes, family history of CDVs, and family history of HPTN.
9	Fan et al. (35)	US	Cross-Sectional	2023	FLI > 60	DII:45 24 h- recall	19,536		Categorical (T4: T1)	OR: 1.59 [95% CI: 1.31 to 1.94]	Age, sex, BMI, physical activity, ethnicity, education level, and comorbidities.
10	Petermann-Rocha et al., (37)	UK	Cohort	2006–2010	MRI-based liver fat/Inpatient diagnosis	E-DII: 18 web-based 24-h dietary assessment tool	171,544	−3.68 to 1.01	Categorical (highest level vs. lowest level)	HR: 1.19 [95% CI: 1.03 to 1.38]	Age, sex, education level, physical activity, smoking, and energy intake.
11	Zhang et al. (36)	China	Cohort	2013–1,019	Ultrasound	Dietary inflammatory potential score: 15 food groups/FFQ	12,877	−1.12 to 1.40	Categorical (quartile 4 vs. quartile 1)	HR = 1.26 (95% CI: 1.13–1.41)	Age, sex, BMI, education level, physical activity, smoking, alcohol consumption, SES, energy intake, family history of the disease, depressive symptoms, anti-inflammatory drug use, and hyperlipidemia.

ALP, alkaline phosphatase; ALT, alanine transaminase; AST, aspartate aminotransferase; BMI, body mass index; CAP, controlled attenuation parameter; CI, confidence interval; CVD, cardiovascular disease; D-AII, dietary anti-inflammation index; DII, dietary inflammatory index; E-DII, energy-adjusted dietary inflammatory index; FFQ, food frequency questionnaire; FLI, fatty liver index; GGT, gamma-glutamyl transferase; HDL, high-density lipoprotein cholesterol; HOMA, homeostasis model assessment; HPTN, hypertension; HR, hazard ratio; HSI, hepatic steatosis index; NAFLD, non-alcoholic fatty liver disease; NS, not specified; OR, odds ratio; SES, socio-economic status; WHR, waist-to-hip ratio. Bold font indicates significant associations or heterogeneity.

### 3.3 Association between DII and NAFLD risk

**Figure 2:**
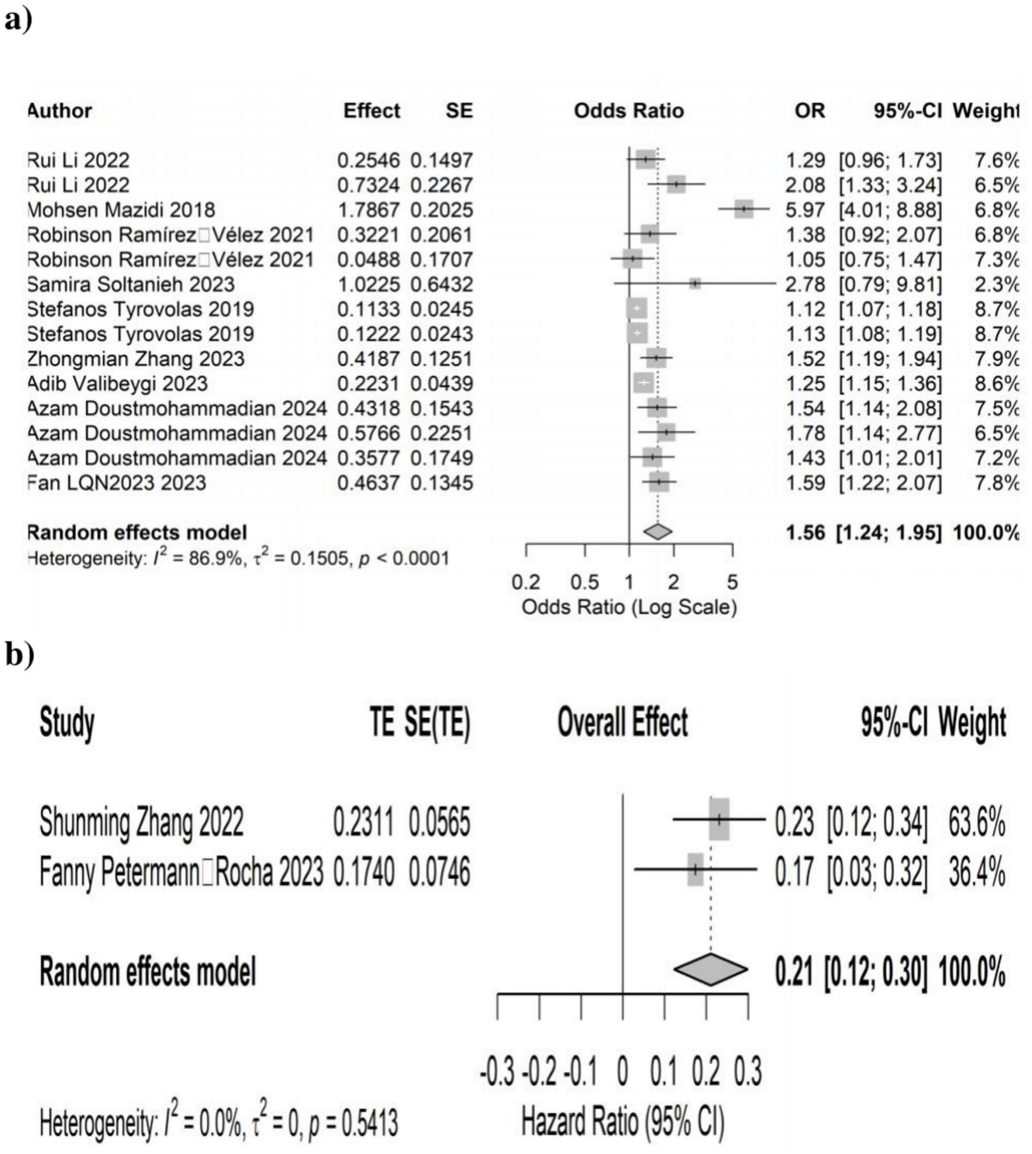
Forest plots illustrates the link between dietary inflammatory index (DII) and the development of non-alcoholic fatty liver disease (NAFLD). **(a)** Cross-sectional studies and **(b)** cohort studies.

Higher DII scores were significantly associated with increased NAFLD risk. In 9 cross-sectional studies (14 effect sizes), the pooled ORs were 1.56 (95% CI: 1.24–1.95; *p* < 0.001), with significant heterogeneity (I^2^ = 86.9%, *p* < 0.0001). In two cohort studies (2 effect sizes), the pooled HR was 0.21 (95% CI: 0.12–0.30; *p* < 0.0001), with no heterogeneity (I^2^ = 0.0%, *p* = 0.54). These findings indicate that diets with greater inflammatory potential increase NAFLD risk across study designs (Figure 2).

### 3.4 Subgroup analyses

Subgroup analyses explored heterogeneity in cross-sectional studies based on BMI, DII definition, sample size, geographical region, age, and NAFLD diagnostic criteria. The significant DII-NAFLD association persisted across most subgroups, including BMI ≥ 25, E-DII, or DII, studies in Asia/Europe, participants <46 years, and studies using HSI with reduced heterogeneity (I^2^ < 50%) in these categories. However, the association was non-significant in studies with smaller sample sizes (<3,042 participants) or used CAP for NAFLD diagnosis (Table 3).

**Table 3:** Results of subgroup analyses for studies evaluating the effect of DII on NAFLD diagnosis in cross-sectional studies.

Subgroup		No. of trials	OR (95% CI)	Subgroup differences (*p*-value)	I^2^ (%)
Total	**-**	14	**1.56 (1.24, 1.95)**	–	**86.9**
BMI	<25	8	**1.69 (1.16, 2.44)**	0.25	**91.7**
	≥25	6	**1.34 (1.18, 1.53)**		34.5
Dietary inflammation assessment	DII	7	**1.22 (1.11, 1.35)**	**0.02**	**65.0**
	E-DII	7	**2.02 (1.33, 3.06)**		**86.2**
Sample size	<3,042 persons	2	1.50 (0.95, 2.35)	0.90	7.0
	≥3,042 persons	12	**1.55 (1.21, 1.98)**		**88.7**
Geographical region	America	7	**1.76 (1.16, 2.66)**	**0.004**	**88.5**
	Asia	5	**1.12 (1.08, 1.16)**		29.1
	Europe	2	**1.12 (1.09, 1.16)**		0
Age	<46 year	2	**1.12 (1.08; 1.16)**	**0.002**	0.0
	≥46 year	12	**1.66 (1.29; 2.15)**		83.8
NAFLD diagnosis instrument	HSI	3	**1.18 (1.03, 1.36)**	0.13	26.6
	FLI	8	**1.75 (1.22, 2.49)**		**92.0**
	CAP	2	1.40 (0.58, 3.35)		**53.3**
	Ultrasound	1	**1.54 (1.13, 2.08)**		–

BMI, body mass index; CI, confidence interval; DII, dietary inflammatory index; NAFLD, non-alcoholic fatty liver disease; FLI, fatty liver index; HSI, hepatic steatosis index; CAP, controlled attenuation parameter. Bold font indicates significant associations or heterogeneity.

### 3.5 Quality and bias assessment

All included studies were of high quality as per the Newcastle–Ottawa Scale (NOS), with cross-sectional studies scoring an average of 7.89 and cohort studies scoring 9 (Table 4). The majority of studies had a low risk of bias, as assessed by ROBINS-I, both overall and for each individual component (e.g., confounding and outcome measurement), except one cross-sectional study (21) with moderate bias in outcome measurement and results reporting (Table 5).

**Table 4:** Quality appraisal of included studies.

Cross-sectional studies								
Studies	Selection (maximum *****)				Comparability (maximum *)	Outcome (maximum***)		
	Representativeness of the cases	Sample size	Non-Response rate	Ascertainment of the screening/surveillance tool	The potential confounders were investigated by subgroup analysis or multivariable analysis	Assessment of outcome	Statistical test	Total scores (maximum 9)
Doustmohammadian et al. (34)	1	1	0	2	1	2	1	8
Mazidi et al. (52)	1	1	0	2	1	2	1	8
Ramírez-Vélez et al. (20)	1	1	0	2	1	2	1	8
Rui and Lin (18)	1	1	0	2	1	2	1	8
Soltanieh et al., 2023 (21)	1	1	0	2	1	1	1	7
Tyrovolas et al., 2019 (23)	1	1	0	2	1	2	1	8
Zhang et al. (32)	1	1	0	2	1	2	1	8
Valibeygi et al. (33)	1	1	0	2	1	2	1	8
Fan et al. (35)	1	1	0	2	1	2	1	8

Cohort studies									
	Selection (maximum ****)				Comparability (maximum **)	Outcome (maximum***)			
	Representativeness of the exposed cohort	Selection of the non-exposed cohort	Ascertainment of exposure	Demonstration that the outcome of interest was not present at the start of study	Comparability of cohort on the basis of the design or analysis	Assessment of outcome	Was the follow-up long enough for the outcome to occur?	Adequacy of follow-up of cohorts	Total scores (maximum 9)
Petermann-Rocha et al. (37)	1	1	1	1	2	1	1	1	9
Zhang et al. (36)	1	1	1	1	2	1	1	1	9

* Denotes the scoring of one star; ** represents the scoring of two stars, which is also the maximum scoring for the comparability domain; *** represents the scoring of three stars and the. Maximum scoring for the outcome domain; **** represents the scoring of four stars and the maximum scoring for the selection domain. The total number of stars awarded to a study indicated its quality, either low (≤3 stars), moderate (4–6 stars), or high (≥7 stars).

**Table 5:** Risk of bias in included studies according to ROBINS-I.

Risk of bias domains								Overall risk of bias
References	Confounding	Selection of participants	Exposure classification	Misclassification	Missing data	Outcome measurement	Reporting of results	
Doustmohammadian et al. (34)	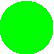	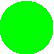	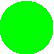	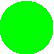	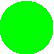	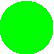	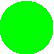	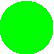
Fan (35)	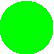	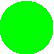	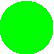	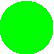	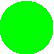	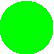	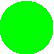	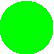
Mazidi et al. (52)	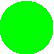	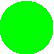	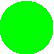	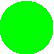	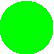	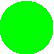	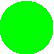	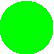
Petermann-Rocha et al. (37)	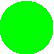	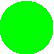	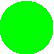	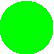	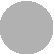	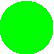	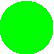	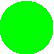
Ramírez-Vélez et al. (20)	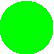	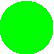	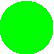	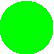	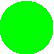	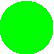	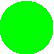	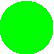
Rui and Lin (18)	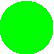	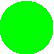	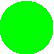	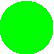	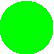	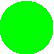	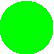	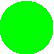
Soltanieh et al. (21)	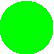	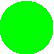	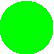	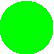	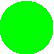	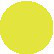	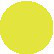	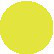
Tyrovolas et al. (23)	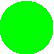	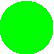	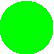	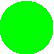	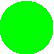	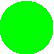	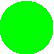	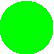
Valibeygi et al. (33)	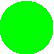	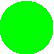	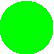	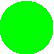	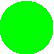	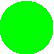	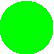	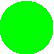
Zhang et al., (32)	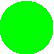	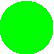	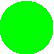	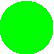	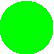	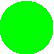	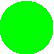	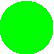
Zhang et al. (36)	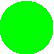	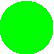	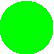	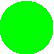	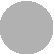	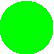	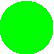	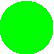

No information on risk of bias 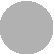; low risk of bias 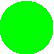; moderate risk of bias 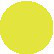; serious risk of bias 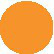; critical risk of bias 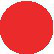. ROBINS-I, Risk of Bias in Non-randomized Studies of Interventions.

### 3.6 Evidence certainty

Based on the GRADE framework, all outcomes were rated “very low” certainty (Table 1). In cross-sectional studies, evidence was downgraded due to inconsistency (13 effect sizes; 8 for FLI, 3 for HSI, and 2 for CAP), publication bias (8 effect sizes; FLI), imprecision (2 effect sizes; CAP), and indirectness (1 effect size; liver ultrasonography). In cohort studies, indirectness was the primary factor.

## 4 Discussion

Our meta-analysis of observational studies demonstrated a significant association between high DII scores and increased NAFLD risk, with a pooled OR of 1.56 (95% CI: 1.24–1.95; *p* < 0.001) in cross-sectional studies and a HR of 0.21 (95% CI: 0.12–0.30; *p* < 0.0001) in cohort studies. These findings, which are built on Qianwen Zhao’s meta-analysis (22) by including four additional high-quality observational studies (18, 20, 23, 24), confirm that diets with greater inflammatory potential are linked to a higher likelihood of NAFLD. This association underscores the role of dietary composition in liver health and the potential of anti-inflammatory diets to mitigate NAFLD risk (38).

Several mechanisms can explain the link between high DII scores and NAFLD. Pro-inflammatory diets, rich in refined carbohydrates, saturated fats, and processed foods, elevate cytokines such as TNF-*α*, IL-6, and CRP, promoting hepatic inflammation and fibrosis (39, 40). These diets also increase oxidative stress through reactive oxygen species (ROS), causing lipid peroxidation and hepatocyte injury (41, 42). Additionally, high DII diets exacerbate insulin resistance, impairing lipid metabolism and driving hepatic fat accumulation (43). Gut microbiota dysbiosis, induced by pro-inflammatory foods, increases intestinal permeability and systemic inflammation via endotoxins such as lipopolysaccharides (LPS) (44), further contributing to NAFLD (45, 46). Imbalanced adipokines, such as reduced adiponectin and elevated leptin, also contribute to hepatic steatosis and inflammation (47–51). These interconnected pathways highlight the multifaceted role of dietary inflammation in NAFLD pathogenesis.

Despite the significant association, substantial heterogeneity (I^2^ = 86.9%, *p* < 0.0001) was observed, reflecting differences in study designs, populations, and DII methodologies. Subgroup analyses revealed that the DII-NAFLD association was consistent across BMI, geographical regions, age groups, and DII assessment methods, strengthening the robustness of our findings. However, the association was not statistically significant in studies with smaller sample sizes (<3,042 participants) or those using CAP for NAFLD diagnosis, likely due to limited statistical power or diagnostic variability. These findings suggest that study size and diagnostic methods influence observed associations, warranting cautious interpretation.

Using the GRADE framework, we rated the evidence certainty as “very low” due to inconsistency, publication bias in cross-sectional studies, and indirectness in cohort studies. Inconsistency arose from methodological variations, while publication bias likely inflated effect sizes due to preferential reporting of significant results. Indirectness in cohort studies stemmed from differences between study populations and real-world settings, limiting generalizability. One of the included cohort studies (37) categorized severe NAFLD as hospitalization or death due to NAFLD/NASH, using hospital/death databases, focusing on advanced endpoints, unlike routine diagnostics (e.g., ultrasound, MRI, and CAP). Another cohort (36) used a novel dietary inflammatory potential score, differing from the standardized DII, thereby adding methodological variability. These differences limited the findings’ clinical applicability, contributing to the “very low” GRADE evidence certainty.

Our results suggest that reducing dietary inflammatory potential could be a key strategy for NAFLD prevention, informing public health policies and dietary guidelines (39). However, the “very low” evidence certainty and reliance on observational studies preclude causal inferences.

Moreover, the DII presents inherent limitations that may affect our findings. DII scores rely on self-reported dietary data, such as food frequency questionnaires, which are susceptible to recall bias and misreporting, potentially affecting the accuracy of the observed association with NAFLD risk. Variability in DII food parameters (e.g., 18–45 parameters) across studies reduces score comparability, contributing to substantial heterogeneity. Additionally, the DII’s inflammatory weights, derived from global literature, may not fully account for dietary or cultural differences across populations (e.g., USA, Iran, Greece, UK, and China), limiting its applicability in diverse settings. The reliance on cross-sectional designs in most studies limits causal inferences, particularly since the DII reflects only short-term dietary patterns. Given these limitations, our findings on the DII-NAFLD association require cautious interpretation. Future research should prioritize longitudinal cohort studies and randomized controlled trials to address the DII’s reliance on self-reported data and cross-sectional limitations, standardize DII assessment and NAFLD diagnostics across diverse populations, and reduce publication bias through comprehensive reporting. Addressing these issues strengthens the evidence base, thereby supporting targeted interventions for NAFLD prevention and management.

## 5 Conclusion

This meta-analysis, integrating cross-sectional and cohort studies, confirms that higher DII scores are associated with increased NAFLD risk, suggesting that anti-inflammatory diets can help prevent NAFLD. However, the systematic application of the GRADE framework revealed “very low” certainty of evidence, highlighting methodological limitations. High-quality cohort studies and randomized controlled trials are needed to strengthen the evidence and guide public health strategies.

## Data Availability

The original contributions presented in the study are included in Supplementary material; further inquiries and requests to access the datasets can be directed to the corresponding author AD, doost_mohammadi@yahoo.com.
