# Supplementary material for: Dietary inflammatory index and non-alcoholic fatty liver disease risk: a systematic review and meta-analysis of observational studies
Source: Front Nutr. 2025 Jun 20;12:1596300. doi: 10.3389/fnut.2025.1596300 (PMC12231496; doi:10.3389/fnut.2025.1596300)
Supplement: Supplementary file 1 [file Table_1.docx]

**Supplementary Materials**

**List of Contents**

**Table S1. Preferred Reporting Items for Systematic Reviews and Meta-Analyses (PRISMA) 2020 Checklist**

Table S2. Excluded studies with reason

**Table S3. Database search strategy for identifying potentially eligible studies**

**Table S1. Preferred Reporting Items for Systematic Reviews and Meta-Analyses (PRISMA) 2020 Checklist**

| **Section and Topic** | **Item #** | **Checklist item** | **Location where item is reported** |
| --- | --- | --- | --- |
| **TITLE** | | |  |
| Title | 1 | Identify the report as a systematic review. | Title page |
| **ABSTRACT** | | |  |
| Abstract | 2 | See the PRISMA 2020 for Abstracts checklist. | Abstract |
| **INTRODUCTION** | | |  |
| Rationale | 3 | Describe the rationale for the review in the context of existing knowledge. | Introduction |
| Objectives | 4 | Provide an explicit statement of the objective(s) or question(s) the review addresses. | Introduction/ last paragraph |
| **METHODS** | | |  |
| Eligibility criteria | 5 | Specify the inclusion and exclusion criteria for the review and how studies were grouped for the syntheses. | Methods/ 1.2. Study eligibility criteria |
| Information sources | 6 | Specify all databases, registers, websites, organisations, reference lists and other sources searched or consulted to identify studies. Specify the date when each source was last searched or consulted. | Methods/ 1.1.Search strategy |
| Search strategy | 7 | Present the full search strategies for all databases, registers and websites, including any filters and limits used. | Methods/ 1.1.Search strategy Table S3 |
| Selection process | 8 | Specify the methods used to decide whether a study met the inclusion criteria of the review, including how many reviewers screened each record and each report retrieved, whether they worked independently, and if applicable, details of automation tools used in the process. | Methods/ 1.1.Search strategy |
| Data collection process | 9 | Specify the methods used to collect data from reports, including how many reviewers collected data from each report, whether they worked independently, any processes for obtaining or confirming data from study investigators, and if applicable, details of automation tools used in the process. | Methods/ 1.3. Data extraction |
| Data items | 10a | List and define all outcomes for which data were sought. Specify whether all results that were compatible with each outcome domain in each study were sought (e.g. for all measures, time points, analyses), and if not, the methods used to decide which results to collect. | Methods/ 1.3. Data extraction/ Table 1 |
|  | 10b | List and define all other variables for which data were sought (e.g. participant and intervention characteristics, funding sources). Describe any assumptions made about any missing or unclear information. | Table 1 |
| Study risk of bias assessment | 11 | Specify the methods used to assess risk of bias in the included studies, including details of the tool(s) used, how many reviewers assessed each study and whether they worked independently, and if applicable, details of automation tools used in the process. | Methods/ 1.4.Quality and risk of bias assessment/ Table 4 |
| Effect measures | 12 | Specify for each outcome the effect measure(s) (e.g. risk ratio, mean difference) used in the synthesis or presentation of results. | Methods/ 1.6.Statistical analysis |
| Synthesis methods | 13a | Describe the processes used to decide which studies were eligible for each synthesis (e.g. tabulating the study intervention characteristics and comparing against the planned groups for each synthesis (item #5)). | Methods/ 1.6.Statistical analysis |
|  | 13b | Describe any methods required to prepare the data for presentation or synthesis, such as handling of missing summary statistics, or data conversions. | Methods/ 1.6.Statistical analysis |
|  | 13c | Describe any methods used to tabulate or visually display results of individual studies and syntheses. | Methods/ 1.6.Statistical analysis |
|  | 13d | Describe any methods used to synthesize results and provide a rationale for the choice(s). If meta-analysis was performed, describe the model(s), method(s) to identify the presence and extent of statistical heterogeneity, and software package(s) used. | Methods/ 1.6.Statistical analysis |
|  | 13e | Describe any methods used to explore possible causes of heterogeneity among study results (e.g. subgroup analysis, meta-regression). | Methods/ 1.6.Statistical analysis |
|  | 13f | Describe any sensitivity analyses conducted to assess robustness of the synthesized results. | Not specified |
| Reporting bias assessment | 14 | Describe any methods used to assess risk of bias due to missing results in a synthesis (arising from reporting biases). | Methods/ 1.4.Quality and risk of bias assessment/ Table 4 |
| Certainty assessment | 15 | Describe any methods used to assess certainty (or confidence) in the body of evidence for an outcome. | Methods/ 1.5. Evaluating the certainty of the evidence/ Table 5 |
| **RESULTS** | | |  |
| Study selection | 16a | Describe the results of the search and selection process, from the number of records identified in the search to the number of studies included in the review, ideally using a flow diagram. | Results/ 2.1.Literature Search and Included Studies/ Figure1 |
|  | 16b | Cite studies that might appear to meet the inclusion criteria, but which were excluded, and explain why they were excluded. | Table S2 |
| Study characteristics | 17 | Cite each included study and present its characteristics. | Results/ 2.2.Study characteristics/ Table 1 |
| Risk of bias in studies | 18 | Present assessments of risk of bias for each included study. | Results/ 2.5. Quality and Bias Assessment/ Table 3 |
| Results of individual studies | 19 | For all outcomes, present, for each study: (a) summary statistics for each group (where appropriate) and (b) an effect estimate and its precision (e.g. confidence/credible interval), ideally using structured tables or plots. | Figure 2/ Table 2 |
| Results of syntheses | 20a | For each synthesis, briefly summarise the characteristics and risk of bias among contributing studies. | Results/ 2.5. Quality and Bias Assessment/ Table3 and Table 4 |
|  | 20b | Present results of all statistical syntheses conducted. If meta-analysis was done, present for each the summary estimate and its precision (e.g. confidence/credible interval) and measures of statistical heterogeneity. If comparing groups, describe the direction of the effect. | Results/ 2.3. Association Between DII and NAFLD Risk/ 2.4. Subgroup Analyses/ Figure 2/Table 2 |
|  | 20c | Present results of all investigations of possible causes of heterogeneity among study results. | Results/ 2.3. Association Between DII and NAFLD Risk/ 2.4. Subgroup Analyses/ Figure 2/Table 2 |
|  | 20d | Present results of all sensitivity analyses conducted to assess the robustness of the synthesized results. | Not specified |
| Reporting biases | 21 | Present assessments of risk of bias due to missing results (arising from reporting biases) for each synthesis assessed. | Results/ 2.5. Quality and Bias Assessment/ Table 4 |
| Certainty of evidence | 22 | Present assessments of certainty (or confidence) in the body of evidence for each outcome assessed. | Results/ 2.5. Evaluating the certainty of the evidence/ Table 5 |
| **DISCUSSION** | | |  |
| Discussion | 23a | Provide a general interpretation of the results in the context of other evidence. | Discussion/ First paragraph |
|  | 23b | Discuss any limitations of the evidence included in the review. | Discussion/ Last paragraph |
|  | 23c | Discuss any limitations of the review processes used. | Discussion/ Last paragraph |
|  | 23d | Discuss implications of the results for practice, policy, and future research. | Discussion/ Last paragraph |
| **OTHER INFORMATION** | | |  |
| Registration and protocol | 24a | Provide registration information for the review, including register name and registration number, or state that the review was not registered. | Methods/ 1.1. Search strategy |
|  | 24b | Indicate where the review protocol can be accessed, or state that a protocol was not prepared. | Methods/ 1.1. Search strategy |
|  | 24c | Describe and explain any amendments to information provided at registration or in the protocol. | Not specified |
| Support | 25 | Describe sources of financial or non-financial support for the review, and the role of the funders or sponsors in the review. | Author contributions/  Funding |
| Competing interests | 26 | Declare any competing interests of review authors. | Conflict of interest |
| Availability of data, code and other materials | 27 | Report which of the following are publicly available and where they can be found: template data collection forms; data extracted from included studies; data used for all analyses; analytic code; any other materials used in the review. | Availability of data and materials |

*From:*  Page MJ, McKenzie JE, Bossuyt PM, Boutron I, Hoffmann TC, Mulrow CD, et al. The PRISMA 2020 statement: an updated guideline for reporting systematic reviews. BMJ 2021;372:n71. doi: 10.1136/bmj.n71

For more information, visit: <http://www.prisma-statement.org/>

Table S2. Excluded studies with reason

| N | Study | Reason for exclusion |
| --- | --- | --- |
|  | Abdallah et al, 2023 | Irrelevant study design (a systematic review) |
|  | Abenavoli et al, 2015 | Irrelevant exposure and study design (a review) |
|  | Abenavoli et al, 2019 | Irrelevant study design (a review/ no observational studies) |
|  | Bullón-Vela et al, 2020 | Irrelevant exposure and study design (a review) |
|  | Bullón-Vela et al, 2020 | Irrelevant exposure (The DII index was not applied) |
|  | Cernea et al, 2023 | Irrelevant exposure and study design (a review) |
|  | Hong et al, 2016 | Irrelevant exposure (focus was on food item instead of dietary pattern DII) |
|  | Cantero et al, 2007 | Irrelevant outcome (multivariable-adjusted hazard ratio or odds ratio (OR) of NAFLD for the highest DII score (pro-inflammatory diet) vs. the lowest DII score (anti-inflammatory diet) was not provided) |
|  | Mitra Darbandi, 2021(23) | Irrelevant outcome (multivariable-adjusted hazard ratio or odds ratio (OR) of NAFLD for the highest DII score (pro-inflammatory diet) vs. the lowest DII score (anti-inflammatory diet) was not provided) |
|  | Khalatbari-Soltani et al, 2019 | Irrelevant exposure (The DII index was not applied) |
|  | Khalatbari-Soltani et al, 2020 | Irrelevant exposure (The DII index was not applied) |
|  | Koch et a, 2015 | Irrelevant exposure (The DII index was not applied) |
|  | Li et al, 2022 | Irrelevant exposure (The DII index was not applied) |
|  | Lozano, 2021(63) | Irrelevant outcome (multivariable-adjusted hazard ratio or odds ratio (OR), along with their 95% confidence intervals (CIs) were not provided) |
|  | Mahmoodi et al, 2020 | Irrelevant exposure (focus was on food item instead of dietary pattern DII) |
|  | Mazidi et al, 2019 | Irrelevant exposure (The DII index was not applied) |
|  | Mirizzi et al, 2019 | Irrelevant exposure (focus was on food item instead of dietary pattern DII) |
|  | Momeni et al, 2023 | Irrelevant exposure (The DII index was not applied) |
|  | Sadri et al, 2022 | Irrelevant outcome |
|  | Taheri et al. 2022 | Irrelevant outcome (failed based on NAFLD definition) |
|  | Tian et al. 2023 | Irrelevant exposure (the aim was to investigate dietary patterns associated with inflammation and whether such diets were associated with the risk of NAFLD. Inflammatory dietary patterns (IDPs) were used instead of DII/E-DII. |
|  | Zheng et al, 2022 | Irrelevant exposure (The DII index was not applied) |
|  | Zhu et al, 2022 | Irrelevant exposure (The DII index was not applied) |
|  | Farhadnejad et al., 2022(64) | Irrelevant exposure (DII was computed with different method) |
|  | Vahid et al. (2019) | Irrelevant exposure (The DII index was not applied) |

**Table S3. Database search strategy for identifying potentially eligible studies**

| **Database** | **Query** |
| --- | --- |
| **PubMed** | (((("Fatty Liver"[Mesh] OR "Fatty Liver"[tiab] OR "Liver, Fatty"[tiab] OR "Steatohepatitis"[tiab] OR "Steatosis of Liver"[tiab] OR "Visceral Steatosis"[tiab] OR "Steatosis, Visceral"[tiab] OR "Liver Steatosis"[tiab] OR "Liver Steatoses"[tiab] OR "Steatosis, Liver"[tiab] OR "Non-alcoholic Fatty Liver Disease"[Mesh] OR "NAFLD"[tiab] OR "Nonalcoholic Fatty Livers"[tiab] OR "Fatty Liver" OR "Liver, Fatty" OR "Steatohepatitis" OR "Steatosis of Liver" OR "Visceral Steatosis" OR "Steatosis, Visceral" OR "Liver Steatosis" OR "Liver Steatoses" OR "Steatosis, Liver" OR "Non-alcoholic Fatty Liver Disease" OR "NAFLD" OR "Nonalcoholic Fatty Livers") AND ("Diet"[Mesh] OR "Diet"[tiab] OR "Diets"[tiab] OR "Inflammation"[Mesh] OR "Inflammation"[tiab] OR "Inflammations"[tiab] OR "Innate Inflammatory Response"[tiab] OR "Inflammatory Response, Innate"[tiab] OR "Innate Inflammatory Responses"[tiab] OR "dietary inflammatory index"[tiab] OR "dietary score"[tiab] OR "dietary inflammatory potential"[tiab] OR "DII"[tiab] OR "anti-inflammatory"[tiab] OR "Diet" OR "Diets" OR "Inflammation" OR "Inflammations" OR "Innate Inflammatory Response" OR "Inflammatory Response, Innate" OR "Innate Inflammatory Responses" OR "dietary inflammatory index" OR "dietary score" OR "dietary inflammatory potential" OR "DII")) AND ("Cohort Studies"[Mesh] OR "Cohort"[tiab] OR "Concurrent Study"[tiab] OR "Incidence"[tiab] OR "Studies, Concurrent"[tiab] OR "Study, Concurrent"[tiab] OR "Prospective Studies"[Mesh] OR "Prospective Studies"[tiab] OR "Prospective Study"[tiab] OR "Studies, Prospective"[tiab] OR "Study, Prospective"[tiab] OR "Case-Control Studies"[Mesh] OR "Case-Control Studies"[tiab] OR "Case Control Study"[tiab] OR "Case-Comparison Study"[tiab] OR "Case-Comparison Studies"[tiab] OR "Case-Compeer Studies"[tiab] OR "Case-Referent Study"[tiab] OR "Case-Base Study"[tiab] OR "Case Base Studies"[tiab] OR "Studies, Case-Control"[tiab] OR "Study, Case-Control"[tiab] OR "Case-Referent Studies"[tiab] OR "Studies, Nested Case-Control"[tiab] OR "Retrospective Studies"[Mesh] OR "Retrospective Studies"[tiab] OR "Retrospective Study"[tiab] OR "Studies, Retrospective"[tiab] OR "Study, Retrospective"[tiab] OR "Cross-Sectional Studies"[Mesh] OR "Cross-Sectional Studies"[tiab] OR "Cross Sectional Study"[tiab] OR "Cross Sectional Analysis"[tiab] OR "Cross Sectional Analyses"[tiab] OR "Cross Sectional Survey"[tiab] OR "Cross-Sectional Surveys"[tiab] OR "Studies, Cross-Sectional"[tiab] OR "Study, Cross-Sectional"[tiab] OR "Analyses, Cross Sectional"[tiab] OR "Disease Frequency Surveys"[tiab] OR "Survey, Cross-Sectional"[tiab] OR "Surveys, Cross-Sectional"[tiab] OR "Analysis, Cross-Sectional"[tiab] OR "Prevalence Study"[tiab] OR "Prevalence Studies"[tiab] OR "Studies, Prevalence"[tiab] OR "Study, Prevalence"[tiab] OR "Systematic Reviews as Topic" [Mesh] OR "Review, Systematic"[tiab] OR "Systematic-Review"[tiab] OR Review*[tiab] OR "Meta-Analysis as Topic"[Mesh] OR "Data pooling"[tiab] OR "Data poolings"[tiab] OR "Meta-Analysis"[tiab] OR "Meta-Analyses"[tiab] OR "MetaAnalysis"[tiab] OR "MetaAnalyses"[tiab] OR "Cohort Studies" OR "Cohort Study" OR "Cohort" OR "Concurrent Study" OR "Incidence" OR "Studies, Concurrent" OR "Study, Concurrent" OR "Prospective Studies" OR "Prospective Study" OR "Studies, Prospective" OR "Study, Prospective" OR "Case-Control Studies" OR "Case-Control Study" OR "Case Control Study" OR "Case-Comparison Study" OR "Case-Comparison Studies" OR "Case-Compeer Studies" OR "Case-Referent Study" OR "Case-Base Study" OR "Case Base Studies" OR "Studies, Case-Control" OR "Study, Case-Control" OR "Case-Referent Studies" OR "Studies, Nested Case-Control" OR "Retrospective Studies" OR "Retrospective Study" OR "Studies, Retrospective" OR "Study, Retrospective" OR "Cross-Sectional Studies" OR "Cross-Sectional Study" OR "Cross Sectional Study" OR "Cross Sectional Analysis" OR "Cross Sectional Analyses" OR "Cross Sectional Survey" OR "Cross-Sectional Surveys" OR "Studies, Cross-Sectional" OR "Study, Cross-Sectional" OR "Analyses, Cross Sectional" OR "Disease Frequency Surveys" OR "Survey, Cross-Sectional" OR "Surveys, Cross-Sectional" OR "Analysis, Cross-Sectional" OR "Prevalence Study" OR "Prevalence Studies" OR "Studies, Prevalence" OR "Study, Prevalence" OR "Systematic Reviews" OR "Systematic Review" OR "Review, Systematic" OR "Systematic-Review" OR Review* OR "Meta-Analysis" OR "Meta-Analyses" OR "Data pooling" OR "Data poolings" OR "MetaAnalysis" OR "MetaAnalyses")) NOT ("Animals"[Mesh] OR "Rats"[Mesh] OR "Mice"[Mesh] OR "Birds"[Mesh] OR Animal* OR "Rats" OR "Mice" OR "Mouse" OR "Piglet")) |
| **Scopus** | TITLE-ABS-KEY ((("Fatty Liver" OR "Liver, Fatty" OR "Steatohepatitis" OR "Steatosis of Liver" OR "Visceral Steatosis" OR "Steatosis, Visceral" OR "Liver Steatosis" OR "Liver Steatoses" OR "Steatosis, Liver" OR "Non-alcoholic Fatty Liver Disease" OR "NAFLD" OR "Nonalcoholic Fatty Livers") AND ("Diet" OR "Diets" OR "Inflammation" OR "Inflammations" OR "Innate Inflammatory Response" OR "Inflammatory Response, Innate" OR "Innate Inflammatory Responses" OR "dietary inflammatory index" OR "dietary score" OR "dietary inflammatory potential" OR "DII" OR "anti-inflammatory") AND ("Cohort Studies" OR "Cohort Study" OR "Cohort" OR "Concurrent Study" OR "Incidence" OR "Studies, Concurrent" OR "Study, Concurrent" OR "Prospective Studies" OR "Prospective Study" OR "Studies, Prospective" OR "Study, Prospective" OR "Case-Control Studies" OR "Case-Control Study" OR "Case Control Study" OR "Case-Comparison Study" OR "Case-Comparison Studies" OR "Case-Compeer Studies" OR "Case-Referent Study" OR "Case-Base Study" OR "Case Base Studies" OR "Studies, Case-Control" OR "Study, Case-Control" OR "Case-Referent Studies" OR "Studies, Nested Case-Control" OR "Retrospective Studies" OR "Retrospective Study" OR "Studies, Retrospective" OR "Study, Retrospective" OR "Cross-Sectional Studies" OR "Cross-Sectional Study" OR "Cross Sectional Study" OR "Cross Sectional Analysis" OR "Cross Sectional Analyses" OR "Cross Sectional Survey" OR "Cross-Sectional Surveys" OR "Studies, Cross-Sectional" OR "Study, Cross-Sectional" OR "Analyses, Cross Sectional" OR "Disease Frequency Surveys" OR "Survey, Cross-Sectional" OR "Surveys, Cross-Sectional" OR "Analysis, Cross-Sectional" OR "Prevalence Study" OR "Prevalence Studies" OR "Studies, Prevalence" OR "Study, Prevalence" OR "Systematic Reviews" OR "Systematic Review" OR "Review, Systematic" OR "Systematic-Review" OR Review* OR "Meta-Analysis" OR "Meta-Analyses" OR "Data pooling" OR "Data poolings" OR "MetaAnalysis" OR "MetaAnalyses")) AND NOT (Animal* OR "Animals" OR "Rat" OR "Rats" OR "Mouse" OR "Mice" OR "Birds" OR "Piglet")) |
| **Web of Sciences** | TS= ((("Fatty Liver" OR "Liver, Fatty" OR "Steatohepatitis" OR "Steatosis of Liver" OR "Visceral Steatosis" OR "Steatosis, Visceral" OR "Liver Steatosis" OR "Liver Steatoses" OR "Steatosis, Liver" OR "Non-alcoholic Fatty Liver Disease" OR "NAFLD" OR "Nonalcoholic Fatty Livers") AND ("Diet" OR "Diets" OR "Inflammation" OR "Inflammations" OR "Innate Inflammatory Response" OR "Inflammatory Response, Innate" OR "Innate Inflammatory Responses" OR "dietary inflammatory index" OR "dietary score" OR "dietary inflammatory potential" OR "DII" OR "anti-inflammatory") AND ("Cohort Studies" OR "Cohort Study" OR "Cohort" OR "Concurrent Study" OR "Incidence" OR "Studies, Concurrent" OR "Study, Concurrent" OR "Prospective Studies" OR "Prospective Study" OR "Studies, Prospective" OR "Study, Prospective" OR "Case-Control Studies" OR "Case-Control Study" OR "Case Control Study" OR "Case-Comparison Study" OR "Case-Comparison Studies" OR "Case-Compeer Studies" OR "Case-Referent Study" OR "Case-Base Study" OR "Case Base Studies" OR "Studies, Case-Control" OR "Study, Case-Control" OR "Case-Referent Studies" OR "Studies, Nested Case-Control" OR "Retrospective Studies" OR "Retrospective Study" OR "Studies, Retrospective" OR "Study, Retrospective" OR "Cross-Sectional Studies" OR "Cross-Sectional Study" OR "Cross Sectional Study" OR "Cross Sectional Analysis" OR "Cross Sectional Analyses" OR "Cross Sectional Survey" OR "Cross-Sectional Surveys" OR "Studies, Cross-Sectional" OR "Study, Cross-Sectional" OR "Analyses, Cross Sectional" OR "Disease Frequency Surveys" OR "Survey, Cross-Sectional" OR "Surveys, Cross-Sectional" OR "Analysis, Cross-Sectional" OR "Prevalence Study" OR "Prevalence Studies" OR "Studies, Prevalence" OR "Study, Prevalence" OR "Systematic Reviews" OR "Systematic Review" OR "Review, Systematic" OR "Systematic-Review" OR Review* OR "Meta-Analysis" OR "Meta-Analyses" OR "Data pooling" OR "Data poolings" OR "MetaAnalysis" OR "MetaAnalyses")) NOT (Animal* OR "Animals" OR "Rat" OR "Rats" OR "Mouse" OR "Mice" OR "Birds" OR "Piglet")) |
| **Cochrane** | (("Fatty Liver" OR "Liver, Fatty" OR "Steatohepatitis" OR "Steatosis of Liver" OR "Visceral Steatosis" OR "Steatosis, Visceral" OR "Liver Steatosis" OR "Liver Steatoses" OR "Steatosis, Liver" OR **"Non-alcoholic Fatty Liver Disease"** OR "NAFLD" OR "Nonalcoholic Fatty Livers") AND ("Diet" OR "Diets" OR "Inflammation" OR "Inflammations" OR "Innate Inflammatory Response" OR "Inflammatory Response, Innate" OR "Innate Inflammatory Responses" OR "dietary inflammatory index" OR "dietary score" OR "dietary inflammatory potential" OR "DII" OR "anti-inflammatory") AND ("Cohort Studies" OR "Cohort Study" OR "Cohort" OR "Concurrent Study" OR "Incidence" OR "Studies, Concurrent" OR "Study, Concurrent" OR "Prospective Studies" OR "Prospective Study" OR "Studies, Prospective" OR "Study, Prospective" OR "Case-Control Studies" OR "Case-Control Study" OR "Case Control Study" OR "Case-Comparison Study" OR "Case-Comparison Studies" OR "Case-Compeer Studies" OR "Case-Referent Study" OR "Case-Base Study" OR "Case Base Studies" OR "Studies, Case-Control" OR "Study, Case-Control" OR "Case-Referent Studies" OR "Studies, Nested Case-Control" OR "Retrospective Studies" OR "Retrospective Study" OR "Studies, Retrospective" OR "Study, Retrospective" OR "Cross-Sectional Studies" OR "Cross-Sectional Study" OR "Cross Sectional Study" OR "Cross Sectional Analysis" OR "Cross Sectional Analyses" OR "Cross Sectional Survey" OR "Cross-Sectional Surveys" OR "Studies, Cross-Sectional" OR "Study, Cross-Sectional" OR "Analyses, Cross Sectional" OR "Disease Frequency Surveys" OR "Survey, Cross-Sectional" OR "Surveys, Cross-Sectional" OR "Analysis, Cross-Sectional" OR "Prevalence Study" OR "Prevalence Studies" OR "Studies, Prevalence" OR "Study, Prevalence" OR "[Systematic Reviews](https://www.ncbi.nlm.nih.gov/mesh/2028178)" OR "Systematic Review" OR "Review, Systematic" OR "Systematic-Review" OR Review* OR "Meta-Analysis" OR "Meta-Analyses" OR "Data pooling" OR "Data poolings" OR "MetaAnalysis" OR "MetaAnalyses")) |
